# Impact of pleural reconstruction on postoperative outcomes in rib tumor resection: a decade-long retrospective study

**DOI:** 10.3389/fsurg.2024.1473791

**Published:** 2024-10-18

**Authors:** Hao Xie, Bowen Li, Yixin Sun, Lin Ma, Qiang Zhang

**Affiliations:** Department of Thoracic Surgery, Beijing Jishuitan Hospital, Capital Medical University, Beijing, China

**Keywords:** pleural reconstruction, rib compartment tumor resection, postoperative drainage, hospital stay, postoperative complications

## Abstract

**Objective:**

This study aims to evaluate the effects of pleural reconstruction during rib compartment tumor resection surgery on postoperative outcomes, including drainage volume, drainage duration, hospital stay, complications, and pain control.

**Methods:**

A retrospective analysis was conducted on 142 patients who underwent rib compartment tumor resection surgery at Beijing Jishuitan Hospital from January 2013 to October 2023. The patients were divided into two groups: those who received pleural reconstruction and those who did not. Data were collected from hospital medical records and outpatient care records, focusing on postoperative drainage volume, total drainage time, length of hospital stay, complications, and pain scores. Continuous variables were compared using *t*-tests or nonparametric tests, while categorical variables were analyzed using chi-square tests or Fisher's exact tests.

**Results:**

The analysis showed no significant differences between the two groups in terms of postoperative complications and pain thresholds. However, patients who underwent pleural reconstruction had significantly lower postoperative drainage volume (937.74 ± 855.97 vs. 1,595.26 ± 1,054.50 ml, *p* < 0.05), shorter drainage duration (5.5 ± 2.39 vs. 8.43 ± 2.87 days, *p* < 0.05), and reduced length of hospital stay (7.32 ± 3.30 vs. 10.99 ± 6.83 days, *p* < 0.05).

**Conclusion:**

Pleural reconstruction during rib compartment tumor resection surgery reduces postoperative drainage volume, drainage duration, and hospital stay without increasing complications or short-term pain. Further large-scale studies are recommended to validate these findings.

## 1 Introduction

Chest wall resection is a pivotal surgical procedure employed in the treatment of benign or low-grade malignant chest wall tumors, presenting an essential strategy for effective disease control and symptomatic relief (1–3). This surgical intervention entails the removal of tumor-affected regions of the chest wall, which necessitates subsequent reconstruction to restore both the structural and functional integrity of the thorax. The primary goals of chest wall reconstruction include maintaining the stability of the chest wall, ensuring the impermeability of the thorax, and preventing any compromise to the respiratory and circulatory systems.

Reconstruction techniques often involve the use of various materials to address the defects created by resection. Steel plates are commonly used to provide rigid support and mechanical stability to the chest wall. Additionally, polymer-based materials are frequently utilized to replace and mimic the properties of the excised soft tissues, thereby facilitating a more comprehensive and effective repair (4–6). These materials and techniques are selected based on the specific requirements of the chest wall defect and the overall condition of the patient.

A critical component of chest wall reconstruction is pleural reconstruction, which aims to reduce the risk of postoperative complications. Such complications can include lung hernia, where the lung protrudes through a defect in the chest wall; chest infections, which can lead to severe respiratory issues; and long-term pleural effusion, characterized by the accumulation of excess fluid around the lungs. These complications can significantly impair patient recovery, prolong hospital stays, and diminish the quality of life (4–6).

Despite the recognized importance of pleural reconstruction in enhancing postoperative outcomes, there is a notable lack of comprehensive research in this area, both domestically and internationally. The existing literature on pleural reconstruction is relatively limited, leaving clinicians with insufficient evidence to fully understand its benefits and optimal application. This gap in knowledge highlights the necessity for detailed studies that explore the efficacy of pleural reconstruction in improving surgical outcomes for patients undergoing chest wall resection.

This study aims to address this critical gap by conducting a thorough evaluation of pleural reconstruction's impact on postoperative outcomes in patients who have undergone chest wall resection. Specifically, we will examine key postoperative metrics such as drainage volume and duration, incidence of complications, and pain levels. By systematically analyzing these outcomes, we seek to provide robust evidence on whether pleural reconstruction offers significant benefits in terms of reducing postoperative complications, shortening hospital stays, and enhancing overall patient recovery and quality of life. While chest wall resection and subsequent reconstruction are well-established procedures for managing benign and low-grade malignant chest wall tumors, the role of pleural reconstruction remains underexplored. This study endeavors to elucidate the value of pleural reconstruction, providing insights that could inform clinical practice and improve patient outcomes in thoracic surgery. Through rigorous retrospective analysis, we aim to contribute to the body of knowledge on chest wall reconstruction, ultimately supporting better clinical decision-making and patient care.

## 2 Materials and methods

### 2.1 Research subjects

This retrospective study was conducted at Beijing Jishuitan Hospital, encompassing the period from January 2013 to October 2023. It involved patients who underwent chest wall compartment resection for benign or low-grade malignant rib cartilage sarcoma. and the so-called compartment resection, which was defined by us as the removal of 3 cm of both ends of the rib where the rib mass was located, encompassing the adjacent upper and lower intercostal muscles, as well as the adjacent pleura and periosteum on the inner layer of the rib and the outer surface of the rib. This resection was carried out in both groups, and the extent of resection was one segment on one rib. Due to concerns regarding inaccurate biopsy results, we implemented this surgical measure to prevent the spread of the patient's tumor. Subsequently, based on the patient's postoperative paraffin pathology, a decision was made as to whether to conduct a secondary surgical extension of the resection. Baseline data were meticulously collected for all patients, including age, gender, body mass index (BMI), pathological diagnosis, and the extent of intraoperative resection. These baseline data were used to compare and contrast the clinical characteristics and disease severity between the two patient groups.

Patients were divided into two groups based on the availability and use of a biological membrane for pleural reconstruction. Initially, due to the unavailability of this material, patients underwent soft tissue coverage only. However, after the introduction of the biological membrane at our hospital, it was used for pleural suturing, leading to a significant reduction in postoperative drainage. This distinction served as the basis for grouping patients and conducting the retrospective analysis to assess the efficacy of pleural reconstruction.

### 2.2 Inclusion criteria

Patients were eligible for inclusion in the study based on the following criteria:

1) Age 18 years or older at the time of surgery.
2) Pathological confirmation of benign rib tumors or low-grade chondrosarcoma based on surgical specimens.
3) Surgical resection involving the removal of the compartment.

### 2.3 Exclusion criteria

Patients were excluded from the study if they met any of the following conditions:

1) Presence of unstable systemic diseases, such as active infections, tuberculosis, uncontrolled hypertension, or unstable angina.
2) History of previous thoracic surgery or preoperative chest computed tomography (CT) indicating pneumonia or atelectasis.
3) Impaired coagulation function.
4) Requirement to terminate the operation due to unpredictable factors such as significant hemorrhage or severe pleural adhesion.
5) Patients with malignant rib tumors requiring the resection of more than three ribs and surrounding soft tissues, which necessitate rigid reconstruction with titanium alloy plates, were excluded from this study to avoid confounding factors. Similarly, benign tumors involving multiple ribs, where resection would span 2–3 ribs, were also excluded to maintain the consistency and reliability of the study outcomes.

### 2.4 Surgical technique

**Figure 1:**
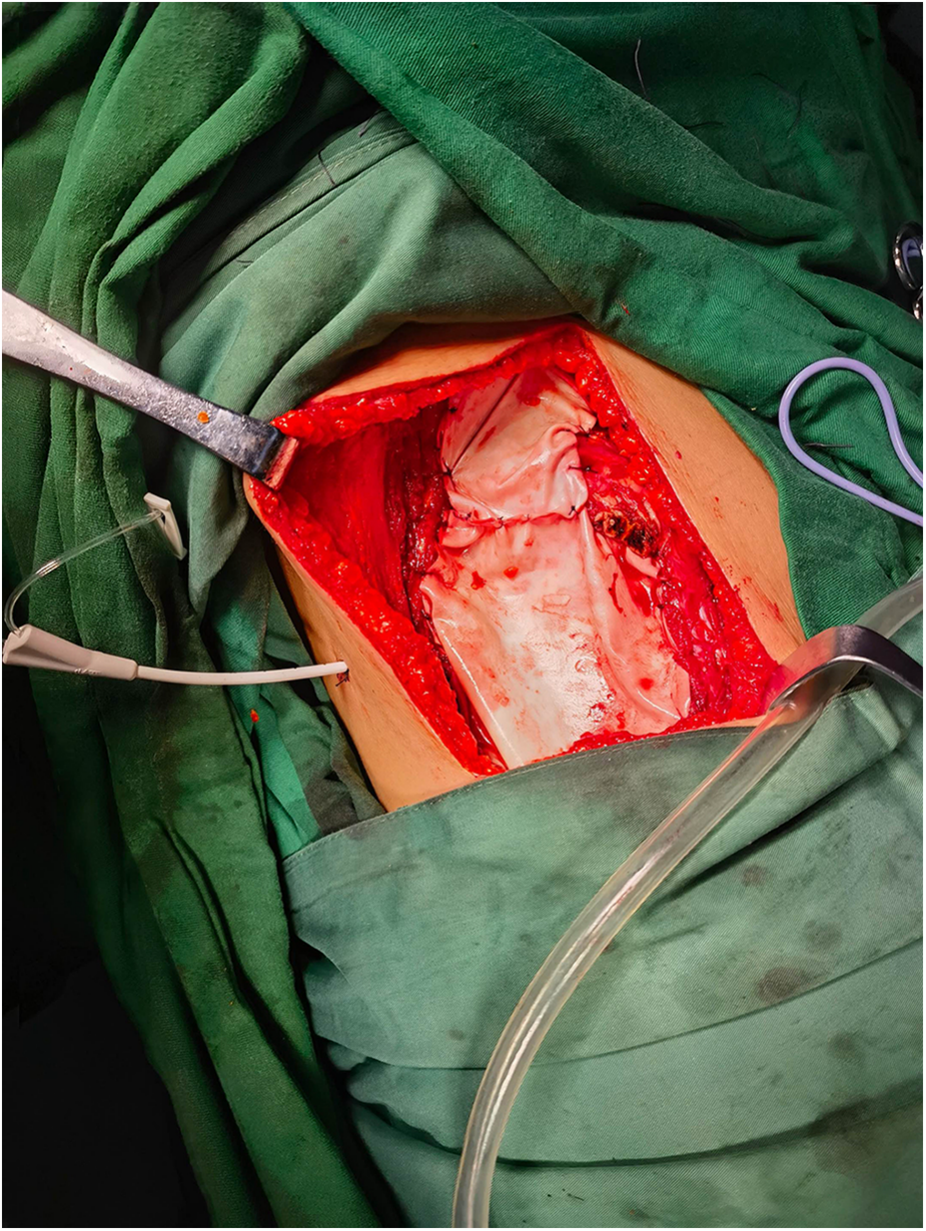
Use of a biological membrane patch for pleural reconstruction.

Patients were placed in a lateral position for the procedure. The surgical approach involved a step-by-step dissection through the muscles to expose the skin lesions and the tumor. Once the tumor was exposed, a rib resection was performed with a margin of at least 3 cm on both ends of the affected rib. This resection included both the upper and lower intercostal muscles to ensure complete removal of the tumor. For the experimental group, the resultant pleural defect was reconstructed using a biological membrane patch, specifically derived from immunogen-removed bovine pericardial tissue. The patch was secured in place with 3-0 absorbable sutures attached to the adjacent ribs and pleura (Figure 1). Following the reconstruction of the pleura, the chest wall muscles were meticulously closed layer by layer. In the control group, the chest wall muscles were closed directly without the use of a pleural reconstruction patch, following a similar step-by-step closure procedure. Drainage tubes were placed in both the chest cavity and the surgical wound to facilitate postoperative fluid management. These tubes were connected to drainage bottles to collect any fluid that accumulated postoperatively. All surgical procedures were performed by the same team of experienced thoracic surgeons to ensure consistency in the surgical technique.

During the surgical procedure, the tumors were solitary, and the resection was carefully planned to include 3 cm on both ends of the affected rib. This resection encompassed the adjacent upper and lower intercostal muscles, the inner layer of the adjacent pleura, and the periosteum on the outer surface of the rib. This comprehensive approach ensured the complete removal of the tumor while maintaining the structural integrity of the chest wall. The size of the chest wall defect created by this resection was thus determined by the extent of the tumor involvement, with the aforementioned 3 cm margin providing an adequate buffer to minimize the risk of local recurrence.

Of note, the term “rib compartment resection” refers to the surgical removal of 3 cm on both ends of the rib where the tumor was located. This procedure included the resection of the adjacent upper and lower intercostal muscles, the inner layer of the pleura, and the periosteum on the outer surface of the rib. The extent of resection was one segment on a single rib for all patients. This approach was adopted to ensure complete tumor removal while minimizing the risk of local recurrence, and subsequent decisions regarding the need for further resection were based on postoperative paraffin pathology results.

Given that the resection involved only one rib, the impact on chest wall stability and the risk of paradoxical respiration were minimal. As a result, titanium alloy plates were not employed for rigid chest wall reconstruction in these cases.

### 2.5 Data collection

Comprehensive data collection was conducted, encompassing both general patient information and detailed surgical and postoperative data. The collected data included demographic information such as age, gender, and body mass index (BMI), as well as specific clinical parameters including pathological diagnosis and the extent of intraoperative resection. During surgery, key metrics such as operation time and intraoperative blood loss were meticulously recorded. Postoperative data collection focused on several critical outcomes:

#### 2.5.1 Postoperative drainage and flow

1) *Postoperative Flow*: The volume of fluid drained from both the chest cavity and surgical wound was recorded.
2) *Postoperative Drainage Duration*: The duration was measured from the time of surgery until the removal of the chest drainage tube and wound drainage tube. Specific criteria for tube removal included the presence of light yellow chest drainage fluid for 3 consecutive days with a volume of less than 100 ml per day and no gas discharge in the chest. The wound drainage tube was removed when the drainage fluid reduced to less than 10 ml per day without pain at the wound site.

#### 2.5.2 Postoperative hospital stay

The length of hospital stay post-surgery was documented for each patient.

#### 2.5.3 Postoperative complications

Detailed records were kept of any complications that occurred, including wound hematoma, encapsulated pleural effusion, pleural effusion, and pulmonary infection. These complications were assessed using chest CT scans.

#### 2.5.4 Pain assessment

Pain levels were evaluated using the Visual Analogue Scale (VAS) at 12, 24, 48, and 72 h post-surgery. The VAS is a widely used quantitative tool for assessing postoperative pain, employing a numerical rating scale from 0 to 10. A score of 0 indicates no pain, 1–3 indicates mild pain, 4–6 indicates moderate pain, and 7–10 indicates severe pain. Pain management included the administration of oral acetaminophen, oxycodone hydrochloride, or injections of pethidine hydrochloride as needed.

### 2.6 Statistical analysis

Data were analyzed using SPSS software (version 26.0, IBM, Armonk, NY, USA). Continuous variables were presented as mean ± standard deviation (SD). Categorical variables were expressed as frequencies and percentages. To compare the differences in continuous variables between the two groups, independent sample *t*-tests were utilized. For the comparison of categorical variables, the chi-square test or Fisher's exact test was applied as appropriate. Statistical significance was set at *p* < 0.05.

### 2.7 Ethical review

This study was conducted as a retrospective observational study and received approval from the Ethics Committee of Beijing Jishuitan Hospital (Approval No. K2024[189]-00). All procedures performed in the study were in accordance with the ethical standards of the institutional and national research committees and with the 1964 Helsinki declaration and its later amendments or comparable ethical standards.

## 3 Results

### 3.1 Baseline data of patients

The baseline characteristics of patients in the observation and control groups were compared, and the relevant data are presented in Table 1. Statistical analysis revealed that there were no significant differences between the two groups in terms of demographic and clinical characteristics, including age, gender, body mass index (BMI), and pathological diagnosis. However, a statistically significant difference was observed in the duration of surgery between the two groups (*p* < 0.05). This indicates that, aside from the duration of the surgical procedure, the two groups were comparable in their baseline characteristics, ensuring a balanced comparison for subsequent analyses. The data provided a solid foundation for further evaluation of postoperative outcomes, ensuring that any observed differences could be attributed to the interventions rather than baseline disparities between the groups.

**Table 1:** Patient demographics and surgical characteristics.

Characteristics	Observation group *n* = 72	The control group *n* = 68	*p* value
Gender (male/female)	43/29	35/17	*p* = 0.249
Age (year)	43.22 ± 14.689	42.22 ± 13.591	*p* = 0.677
Body mass index	23.12 ± 2.16	22.91 ± 1.98	*p* = 0.554
Duration of surgery (hour)	2.02 ± 0.17	1.50 ± 0.10	*p* = 0.000
Blood loss (ml)	50.14 ± 9.96	49.94 ± 6.92	*p* = 0.892
Length of surgical resection (cm)	10.42 ± 2.46	10.09 ± 3.71	*p* = 0.536

In addition to the demographic and clinical characteristics, we also analyzed the histological types of tumors resected in both the observation and control groups. The histological types included fibrous dysplasia, osteochondroma, hemangioma, xanthoma, and aneurysmal bone cysts. Specifically, in the observation group, the distribution was as follows: fibrous dysplasia (36 cases), osteochondroma (1 case), hemangioma (6 cases), xanthoma (2 cases), and aneurysmal bone cyst (5 cases). In the control group, the distribution was: fibrous dysplasia (26 cases), osteochondroma (8 cases), hemangioma (4 cases), xanthoma (3 cases), and aneurysmal bone cyst (4 cases). This detailed pathological information helps in understanding the underlying tumor characteristics in our patient population and provides a comprehensive overview of the baseline tumor histology across the two groups.

### 3.2 Comparison of postoperative drainage effects between the two groups

The analysis of postoperative drainage effects revealed significant differences between the observation group and the control group. Specifically, the total drainage volume, postoperative drainage duration, and length of postoperative hospitalization were significantly lower in the observation group compared to the control group (*p* < 0.05). These findings suggest that pleural reconstruction in the observation group contributed to improved postoperative fluid management and reduced hospital stays. Detailed results are presented in Table 2.

**Table 2:** Comparison of postoperative drainage effects between the two groups.

Postoperative results	The observation group, *n* = 72	The control group *n* = 68	*p* value
Drainage volume (ml)	937.74 ± 855.97	1,595.26 ± 1,054.50	0.000
Drainage duration (day)	5.5 ± 2.39	8.43 ± 2.87	0.000
The postoperative hospitalization time (day)	7.32 ± 3.30	10.99 ± 6.83	0.000

### 3.3 Comparison of postoperative complications between the two groups

The comparison of postoperative complications showed no significant differences between the observation and control groups. This indicates that pleural reconstruction did not increase the risk of complications such as fever, wound infection, pleural effusion, or wound hematoma. The detailed comparison is provided in Table 3.

**Table 3:** Postoperative complications comparison between the two groups.

Postoperative complications	Observation group, *n* = 72	The control group *n* = 68	*p* value
Fever	10	9	0.910
Wound infection	1	3	0.283
Pleural effusion	1	5	0.082
Wound hematoma	1	4	0.152

### 3.4 Comparison of postoperative pain scores between the two groups

Postoperative pain was assessed using the Visual Analogue Scale (VAS) at multiple time points (12, 24, 48, and 72 h post-surgery). The analysis indicated no significant differences in pain scores between the observation and control groups at any of these time points. This suggests that pleural reconstruction did not exacerbate postoperative pain. The detailed pain assessment results are presented in Table 4.

**Table 4:** Comparison of postoperative VAS pain scores between the two groups.

Postoperative pain score	Observation group, *n* = 72	The control group *n* = 68	*p* value
12 h	7.28 ± 0.91	7.40 ± 0.87	*p* = 0.428
24 h	6.10 ± 0.88	6.10 ± 0.85	*p* = 0.969
48 h	5.33 ± 0.93	5.26 ± 0.77	*p* = 0.636
72 h	4.79 ± 0.98	5.06 ± 0.77	*p* = 0.676

These findings collectively demonstrate that pleural reconstruction is effective in reducing postoperative drainage and hospital stay without increasing the risk of complications or pain, thereby potentially enhancing overall postoperative recovery.

## 4 Discussion

Chest wall resection is a critical method for treating benign and low-grade malignant rib tumors. Some surgeons employ an *en bloc* resection technique, also known as rib compartment resection, which involves removing the tumor along with 2–3 cm margins of normal rib tissue and the surrounding muscle, bone fascia, and pleura (7). This comprehensive approach aims to ensure complete tumor excision and reduce the risk of local recurrence (8).

The introduction of new biological materials in clinical practice has significantly impacted chest wall reconstruction. Titanium alloy materials and biological patches are increasingly used to reconstruct chest wall defects, providing the necessary stability (9–11). These materials offer rigid support and are often supplemented by the use of polymer membrane patches for soft tissue repair (12). However, detailed protocols for the hierarchical suturing of these soft tissue patches have not been extensively reported in the literature.

The concept of pleural reconstruction remains underrepresented in academic discussions. There is no consensus among physicians regarding the necessity and methods of chest wall reconstruction. Some believe that defects smaller than 5 cm do not require rigid reconstruction, while larger defects necessitate comprehensive reconstruction strategies. The selection of reconstruction techniques should consider the defect's size, location, and the remaining chest wall structure, potentially combining local and distant free tissue transplantation. The key is to select the technology associated with the minimum incidence of complications (13).

Literature reviews suggest that beyond rigid structure reconstruction, the use of myocutaneous flaps is a primary method for soft tissue repair in chest wall reconstruction (10, 14, 15). These flaps provide robust coverage for the wound, enhancing the stability and integrity of the reconstructed chest wall. Our study contributes to this ongoing discussion by providing evidence that pleural reconstruction can significantly reduce postoperative drainage volume, drainage duration, and hospitalization time without increasing complications or postoperative pain. These findings suggest that incorporating pleural reconstruction into chest wall resection surgeries can enhance patient outcomes and recovery, supporting its broader adoption in clinical practice. Further research with larger sample sizes and longer follow-up periods is necessary to validate these findings and refine the techniques and materials used in pleural reconstruction.

In 2018, G. Sandler and colleagues explored the potential future use of non-rigid materials for chest wall repair. Their study suggests that the future of chest wall reconstruction is likely to involve absorbable semi-rigid patches, biological integrations of decellularized homografts and xenografts, decalcified bone matrix, and bone marrow stromal cells. Additionally, they foresee the use of lab-grown vascularized bone, muscle, and skin based on stem cell grafts developed from the patients themselves (5). However, the authors did not provide detailed insights into the role of pleural reconstruction within this context. The prognosis of patients undergoing pleural reconstruction has not been extensively studied. This research retrospectively examines 10 years of thoracic surgeries involving chest wall tumor excision at Beijing Jishuitan Hospital. The focus is on comparing the short-term prognostic effects of pleural reconstruction vs. no pleural reconstruction, particularly in terms of postoperative drainage.

Our findings indicate that suturing biofilm patches to pleural stumps and reconstructing the pleura significantly affect postoperative fluid dynamics compared to merely covering the pleura with soft tissue after resection. The physiological function of the pleura in secreting and absorbing fluids is compromised after trauma, increasing fluid secretion and reducing absorption (16). Consequently, after pleural resection, the balance of fluid absorption and secretion is disrupted, leading to the accumulation of non-functional exudates in the surgical area. This accumulation raises the risk of atelectasis and wound infection, underscoring the necessity for postoperative drainage.

**Figure 2:**
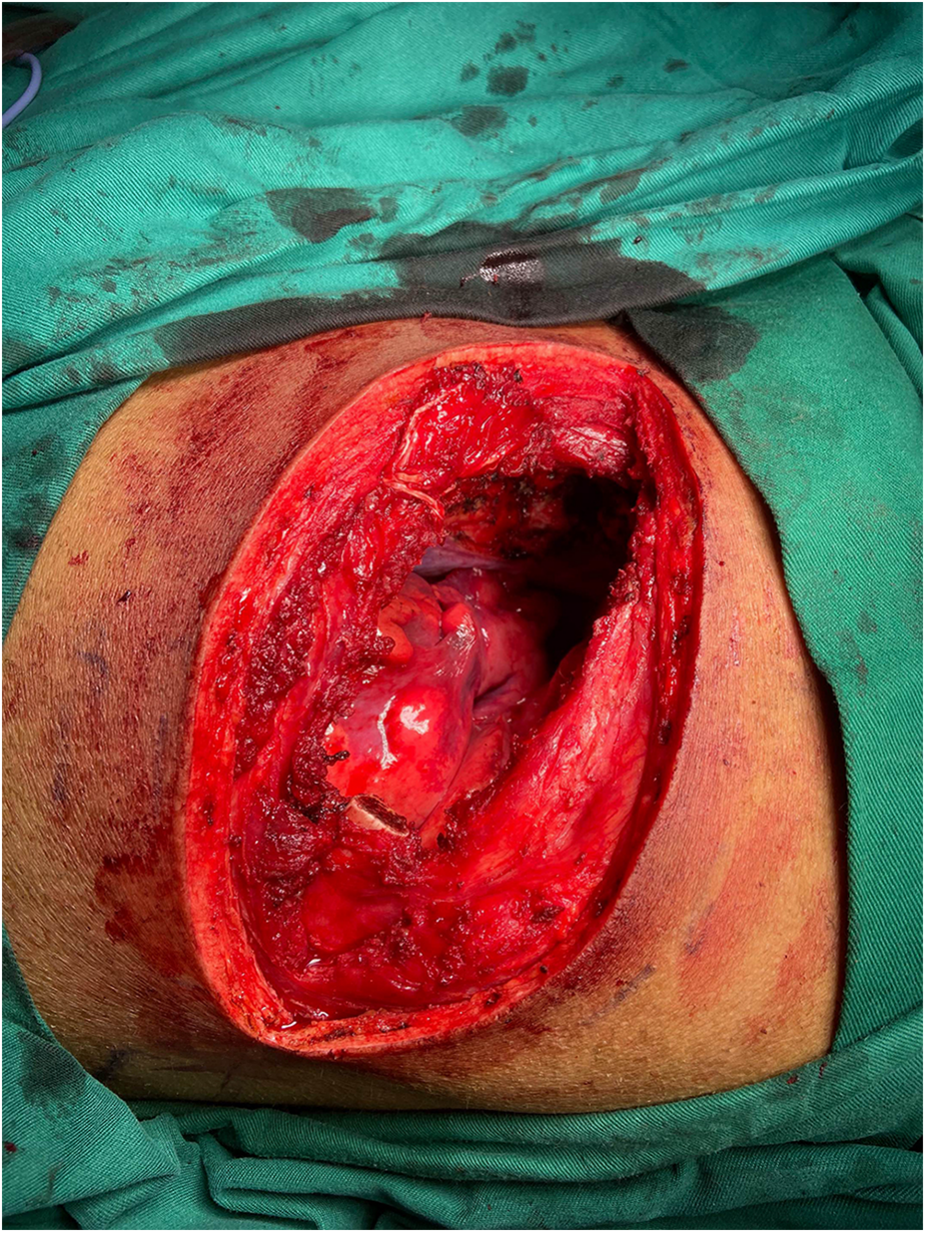
Chest wall defect repair using muscle tissue without pleural reconstruction.

When the remaining chest wall muscles are used to cover the chest cavity without pleural reconstruction (Figure 2), there is a higher likelihood of increased fluid volume due to seepage. Conversely, using biological materials to stitch and repair the pleural stumps can reduce local pleural side leakage, decrease friction between the lung and chest wall muscles, and minimize interference with lung movement during respiration. Additionally, the biological materials help maintain negative pressure within the pleural cavity, preventing lung compression and reducing partial tension during coughing. A relevant study on suturing the mediastinal pleura after esophagectomy found that this approach reduced the incidence of atelectasis, anastomotic leakage, delayed gastric emptying, and the severity of leaks. Importantly, it did not increase the incidence of other complications or mortality related to the operation and postoperative hospitalization (17). This evidence supports the potential benefits of pleural reconstruction in thoracic surgeries, highlighting its role in improving postoperative outcomes and reducing complications. Overall, our study underscores the importance of pleural reconstruction in enhancing patient recovery and reducing postoperative complications, paving the way for further research and potential advancements in thoracic surgical techniques.

The amount of postoperative drainage and the duration of drainage significantly influence a patient's discharge time and overall rehabilitation following chest wall tumor excision surgery. Ensuring the safe and timely removal of the chest tube is increasingly recognized as a critical aspect of postoperative care (12). Studies indicate that larger chest wall defects have a greater adverse impact on respiratory function, leading to more complications and prolonged recovery periods (4). In our study, a clear difference was observed between the observation group and the control group regarding postoperative drainage volume and the length of hospital stay. Patients in the observation group, who underwent pleural reconstruction, experienced shorter drainage times and reduced hospital stays compared to the control group. This suggests that the surgical method of suturing the pleural stump and reconstructing the pleura effectively enhances recovery by minimizing postoperative fluid accumulation and facilitating earlier discharge. Postoperative pain is often a result of nerve stimulation following chest wall resection. Our study found no significant differences in short-term pain levels between the observation and control groups, indicating that the use of biofilm patches for pleural reconstruction does not exacerbate pleural or intercostal nerve irritation. This suggests that pleural reconstruction does not increase postoperative pain and may offer other clinical benefits.

As a retrospective study, this investigation utilized historical data and records to group study subjects, which inherently limits the ability to perform randomization and introduces potential bias. The lack of randomization means the study cannot provide the same level of causal inference and proof as a randomized controlled trial. This is a recognized limitation of retrospective studies. To address this, future research should include prospective randomized controlled trials to validate our findings. Furthermore, the study's short-term follow-up does not allow for the assessment of the long-term benefits and safety of pleural reconstruction. Therefore, additional studies with larger sample sizes and extended follow-up periods are necessary to fully confirm the efficacy and safety of pleural reconstruction techniques. There is also a scarcity of clinical studies comparing the effectiveness of pleural reconstruction vs. no reconstruction in different conditions. This gap limits our understanding of its performance across various clinical scenarios. Rigorous and extensive clinical trials are essential to explore and compare the efficacy of pleural reconstruction in chest wall surgeries. Such research will help in developing evidence-based guidelines and optimizing surgical techniques to improve patient outcomes and enhance the overall quality of postoperative care.

In this study, the material used for pleural reconstruction was a biological membrane patch derived from immunogen-removed bovine pericardial tissue. This material was selected due to its availability and its ability to provide effective reconstruction without significant complications. However, we acknowledge the potential benefits of synthetic materials, such as those made from polytetrafluoroethylene (PTFE) or polypropylene, which are known for their durability and resistance to infection. Currently, synthetic materials were not employed in our study due to supply constraints and the focus on using readily available biological options. Moving forward, we plan to explore the use of synthetic materials in pleural reconstruction, particularly if they demonstrate superior patient outcomes in terms of reducing postoperative complications and enhancing recovery. Future research will involve a comparative analysis of these materials to determine the most effective options for pleural reconstruction in thoracic surgery.

## 5 Conclusion

Our study underscores the significant benefits of incorporating pleural reconstruction in chest wall surgery. The findings demonstrate that pleural reconstruction can effectively reduce postoperative drainage volume, shorten the duration of drainage, and decrease the length of hospital stay. These advantages are achieved without compromising the efficacy when compared to conventional chest tube methods. While pleural reconstruction shows promise, it is not without potential drawbacks. The procedure may be associated with complications such as hematoma and pleural effusion, and it generally requires a longer operative time compared to approaches that do not include pleural reconstruction. Despite these challenges, the benefits of pleural reconstruction, including improved postoperative outcomes and enhanced patient recovery, make it a compelling option in chest wall reconstruction surgery. The selection of the appropriate pleural reconstruction method and materials should be tailored to the specific clinical circumstances of each patient. In this study, the use of immunogen-removed bovine pericardial tissue proved effective for pleural reconstruction. However, the results highlight the need for further research. Larger studies with extended follow-up periods are essential to validate our findings and to comprehensively assess the long-term efficacy and safety of pleural reconstruction techniques. Future research should aim to explore various reconstruction materials and techniques in diverse clinical settings to optimize surgical outcomes. Rigorous and extensive clinical trials are necessary to develop evidence-based guidelines that will inform clinical practice and enhance the overall quality of care for patients undergoing chest wall reconstruction surgery.

## Data Availability

The original contributions presented in the study are included in the article/Supplementary Material, further inquiries can be directed to the corresponding author.
